# Manipulating Hubbard-type Coulomb blockade effect of metallic wires embedded in an insulator

**DOI:** 10.1093/nsr/nwac210

**Published:** 2022-10-04

**Authors:** Xing Yang, Zhao-Long Gu, Huimin Wang, Jing-Jing Xian, Sheng Meng, Naoto Nagaosa, Wen-Hao Zhang, Hai-Wen Liu, Zi-Heng Ling, Kai Fan, Zhi-Mo Zhang, Le Qin, Zhi-Hao Zhang, Yan Liang, Jian-Xin Li, Ying-Shuang Fu

**Affiliations:** School of Physics and Wuhan National High Magnetic Field Center, Huazhong University of Science and Technology, Wuhan 430074, China; National Laboratory of Solid State Microstructures and Department of Physics, Nanjing University, Nanjing 210093, China; Beijing National Laboratory for Condensed Matter Physics and Institute of Physics, Chinese Academy of Sciences, Beijing 100190, China; School of Physics and Wuhan National High Magnetic Field Center, Huazhong University of Science and Technology, Wuhan 430074, China; Beijing National Laboratory for Condensed Matter Physics and Institute of Physics, Chinese Academy of Sciences, Beijing 100190, China; RIKEN Center for Emergent Matter Science (CEMS), Wako 351-0198, Japan; Department of Applied Physics, University of Tokyo, Tokyo 113-8656, Japan; School of Physics and Wuhan National High Magnetic Field Center, Huazhong University of Science and Technology, Wuhan 430074, China; Center for Advanced Quantum Studies, Department of Physics, Beijing Normal University, Beijing 100875, China; School of Physics and Wuhan National High Magnetic Field Center, Huazhong University of Science and Technology, Wuhan 430074, China; School of Physics and Wuhan National High Magnetic Field Center, Huazhong University of Science and Technology, Wuhan 430074, China; School of Physics and Wuhan National High Magnetic Field Center, Huazhong University of Science and Technology, Wuhan 430074, China; School of Physics and Wuhan National High Magnetic Field Center, Huazhong University of Science and Technology, Wuhan 430074, China; School of Physics and Wuhan National High Magnetic Field Center, Huazhong University of Science and Technology, Wuhan 430074, China; School of Physics and Wuhan National High Magnetic Field Center, Huazhong University of Science and Technology, Wuhan 430074, China; National Laboratory of Solid State Microstructures and Department of Physics, Nanjing University, Nanjing 210093, China; Collaborative Innovation Center of Advanced Microstructures, Nanjing University, Nanjing 210093, China; School of Physics and Wuhan National High Magnetic Field Center, Huazhong University of Science and Technology, Wuhan 430074, China; Hubei Key Laboratory of Gravitation and Quantum Physics, Huazhong University of Science and Technology, Wuhan 430074, China; Wuhan Institute of Quantum Technology, Wuhan 430206, China

**Keywords:** STM, one-dimensional, Coulomb effect, electron correlation, Hubbard

## Abstract

Correlated states have emerged in low-dimensional systems owing to enhanced Coulomb interactions. Elucidating these states requires atomic-scale characterization and delicate control capabilities. Herein, spectroscopic imaging-scanning tunneling microscopy was employed to investigate the correlated states residing in 1D electrons of the monolayer and bilayer MoSe_2_ mirror twin boundary (MTB). The Coulomb energies, determined by the wire length, drive the MTB into two types of ground states with distinct respective out-of-phase and in-phase charge orders. The two ground states can be reversibly converted through a metastable zero-energy state with *in situ* voltage pulses, which tune the electron filling of the MTB via a polaronic process, substantiated by first-principles calculations. Our Hubbard model calculation with an exact diagonalization method reveals the ground states as correlated insulators from an on-site U-originated Coulomb interaction, dubbed the Hubbard-type Coulomb blockade effect. Our study lays a foundation for understanding and tailoring correlated physics in complex systems.

## Teaser

A new mysterious correlated state, dubbed Hubbard-type Coulomb blockade effect, is uncovered in metallic wires embedded in MoSe_2_ ultrathin films, which can be delicately controlled in-situ at atomic scale.

## INTRODUCTION

In reduced dimensions, the screening effect is suppressed, resulting in enhanced Coulomb interactions. Owing to their structural simplicity, low-dimensional systems provide model platforms for studying correlated physics [1–5]. In particular, 1D electrons are endowed with divergent electron susceptibility owing to their perfect Fermi surface nesting, making them susceptible to interactions [6,7]. These are exemplified by the Peierls-type charge density wave (CDW) originating from the periodic lattice distortion induced by electron–phonon interactions [8–10]. They are also exemplified by the fractionalized spin-charge separation in the Tomonaga Luttinger liquid (TLL) as a result of electron–electron interactions [11,12]. Thus, interesting 1D electrons serve as paradigmatic systems for the investigation of emergent correlated states [13].

Actual 1D systems are subject to quantum fluctuations that profoundly influence correlated states [14–16]. Inter-chain coupling can suppress such an influence; however, it also introduces complications to the system. Recent studies have identified a new 1D metallic system residing in the mirror twin boundary (MTB) of transition metal dichacogenides [17,18]. This system is devoid of inter-chain coupling and has negligible quantum fluctuations because of its embedment inside the bulk insulating matrix [16,18]. These properties render it an ideal candidate for studying correlated physics in 1D systems.

In correlated phases, the electron-filling adjustment is not only decisive for pinning down the nature of the ground states, but also engages with Coulomb interactions for tuning phase transitions. It is desirable to investigate the evolution of the correlated phases with the *in situ* response of electron filling. Conventional methods of carrier doping inevitably introduce disorders into the system. In low-dimensional systems, electric gating acts as another means of tuning electron filling. Recent achievements in such phase transitions between Mott-like insulators, superconductors and topological orders have been realized in twisted magic-angle graphene superlattices [19–21]. However, the electric-gating method requires sophisticated procedures for device fabrication.

In this study, we characterize the correlated states in 1D MTB wires and demonstrate their *in situ* manipulation using spectroscopic imaging-scanning tunneling microscopy (SI-STM) at 4 K. MTBs are driven into two types of correlated insulating states arising from the dubbed Hubbard-type Coulomb blockade (CB) effect. The two ground states can be switched controllably with voltage pulses, which tune the electron filling of the MTB. Our study clarifies the nature of the correlated ground states and demonstrates the feasibility of local control.

## RESULTS AND DISCUSSION

**Figure 1.**
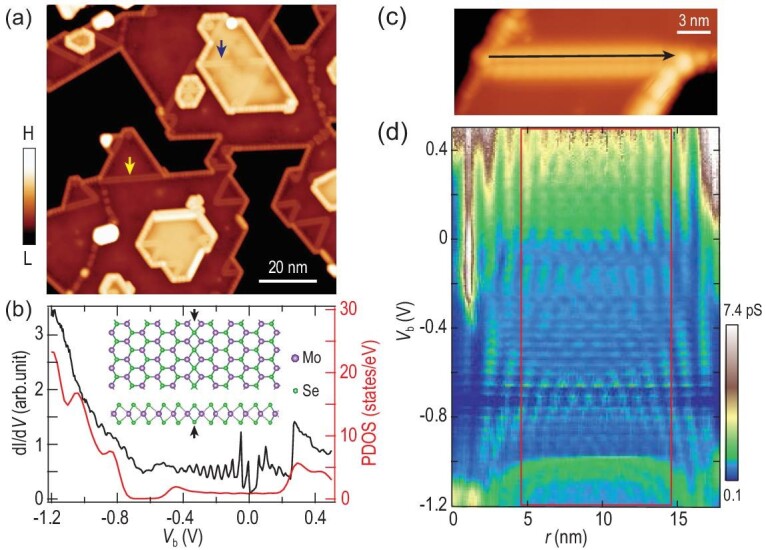
Topography and electronic structure of MoSe_2_ MTB. (a) STM image (*V*_b_ = 500 mV, *I*_t_ = 20 pA) of MTB. Exemplified monolayer and bilayer MTBs are marked with yellow and black arrows, respectively. (b) DFT-calculated partial density of states (PDOS) (red curve) and experimental spectrum (black curve) of an MTB. Inset: Top view and side view of the MTB crystal structure. The black arrows mark the MTB position. The sample bias is labeled as *V*_b_. (c) Magnified STM image (*V*_b_ = 500 mV, *I*_t_ = 10 pA) of a monolayer MTB. (d) Conductance plot (*V*_b_ = 500 mV, *I*_t_ = 100 pA, *V*_mod_ = 3.54 mV) along the black line in (c). The spectrum in (b) is averaged from (d) in the rectangle.

Figure 1a shows the topography of the MTBs, which appears as bright straight-line defects embedded in the monolayer and bilayer MoSe_2_ films on the graphene-covered SiC substrate. Their crystal structure (Fig. 1b, inset) conforms to our atomic-resolution imaging (Note 1 in the Supplementary Material). The line spectra along the wire (Fig. 1c) exhibit discrete levels (Fig. 1d), which are quantum well states (QWSs) due to electron confinement along the finite MTB (Note 2 in the Supplementary Material). The spectrum of the MTB (Fig. 1b) features an abrupt conductance onset peak at 0.27 eV, a series of peaks related to the QWSs and a spectral dip at −0.65 eV. These spectroscopic features are effectively captured by density functional theory (DFT) calculations with an MTB Mo–Mo bond of 2.66 Å. After structural relaxation, the calculated MTB exhibits a stable 1 × 1 structure without any signature of spontaneous distortion, excluding the possibility of the CDW state from the Peierls mechanism. The calculated MTB lattice constant (0.33 nm) agrees with the measured value (0.34 nm).

**Figure 2.**
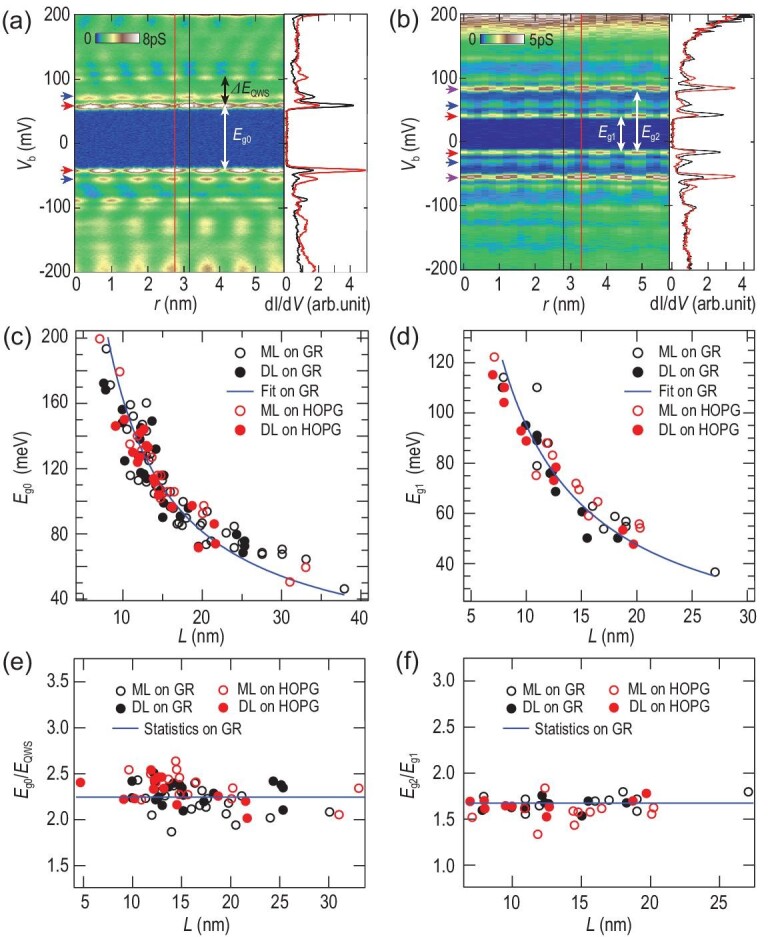
Two types of MTB. (a) and (b) Left: 2D conductance plot [*V*_b_ = 200 mV, *I*_t_ = 100 pA, *V*_mod_ = 1.414 mV (rms)] of the (a) out-of-phase and (b) in-phase MTB. Right: Point spectra extracted along two conductance modulation extrema from the red and black lines. (c) and (d) Statistics showing E*_g_*_0_ and E*_g_*_1_ as a function of *L*. The blue curves are fittings to the data on graphene (GR), yielding ${E}_{g0} = \frac{{1.64 \pm 0.04({\rm{eV}} \cdot {\rm{nm}})}}{L}$ and ${E}_{g1} = \frac{{0.95 \pm 0.03({\rm{eV}} \cdot {\rm{nm)}}}}{L}$. (e) and (f) Statistics showing the ratios of ${E}_{g0}/\Delta {E}_{QWS}$ and ${E}_{g2}/{E}_{g1}$, respectively, whose statistical values on the graphene substrate are marked with blue lines. In (c)–(f), the open (solid) dots mark the corresponding values measured on monolayer (ML) [double-layer (DL)] MTBs, with the black (red) color representing the data on the graphene (HOPG) substrate.

The low-energy states around the Fermi level *E_F_* (Fig. 2a and b) exhibit full gaps and strong peaks at the gap edges (Fig. 2a and b, red arrows), with evidently higher intensities than the QWSs. MTBs of different lengths all exhibit satellite peaks of 14 ± 1 meV spacing, whose intensities follow those of the QWSs and the low-energy peaks (Fig. 2a and b, blue arrows), and have thus been ascribed as phonon peaks previously [18], instead of Coulomb staircases. Interestingly, two types of MTBs are observed. For the Type-1 MTB [Fig. 2(a)], the conductance modulation of its two low-energy peaks is out of phase. The out-of-phase relationship gradually diminishes at the wire ends. Its spectral gap size, ${E}_{g0}$, is uniform throughout the wire and its magnitude is much larger than the energy spacing between the QWSs, $\Delta {E}_{QWS}$. Type-2 MTB (Fig. 2b) has a similar gap, but with substantial differences. First, the gap size ${E}_{g1}$ is close to $\Delta {E}_{QWS}$. Second, the spatial modulation of its low-energy peaks is strictly in phase. Third, the two second low-energy peaks (Fig. 2b, purple arrows) are even more enhanced. We define the energy separation between the first occupied peak and the second unoccupied peak near ${E}_F$ as$\ {E}_{g2}$. Hereinafter, Type-1 and Type-2 MTBs are equivalently designated with their phase relations as the out-of-phase and in-phase states, respectively.

Statistics for >80 MTBs indicate that the ratio between Type-1 and Type-2 wires is ∼4:1. The more abundant Type-1 MTB is possibly related to its larger gap around *E_F_*, which decreases the system energy more effectively. Moreover, the gap sizes of both low-energy states exhibit a pertinent inversely proportional relationship with the wire length, *L* (Fig. 2c and d) [22,23]. A similar relationship exists between $\Delta {E}_{QWS}$ and *L*, which prompts us to evaluate their relations. For the Type-1 MTBs with various lengths, their ratios of ${E}_{g0}/\Delta {E}_{QWS}$ are similar, with statistics of 2.25 ± 0.14 (standard deviation) (Fig. 2e). Similar ratios of ${E}_{g2}/{E}_{g1}$ apply to Type-2 MTBs with 1.68 ± 0.07 (Fig. 2f). The gap sizes of both types of MTBs in monolayer and double-layer MoSe_2_ exhibit identical dependence on *L*. Moreover, no difference is observed between the graphene substrate and highly oriented pyrolytic graphite (HOPG) (Fig. 2c–f).

**Figure 3.**
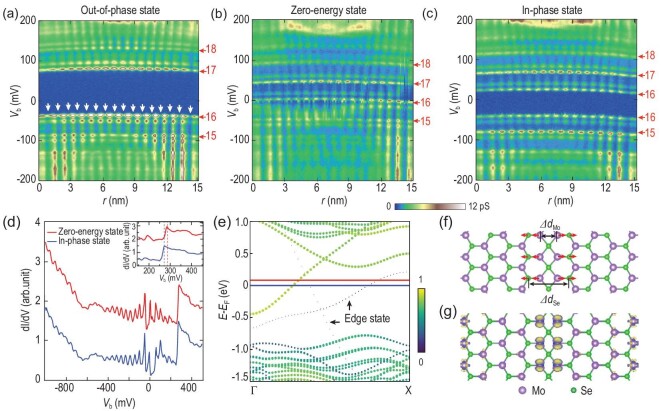
Ground-state transitions of MTB manipulated with tip pulses. (a)–(c) 2D conductance plot [*V*_b_ = 200 mV, *I*_t_ = 100 pA, *V*_mod_ = 1.414 mV (rms)] of the same MTB as shown in Fig. 1c, displaying different ground states. The node numbers for each discrete level are marked in red, and are defined as the numbers of minima in the charge-density modulations of corresponding levels, as exemplified with white arrows in (a). (d) Large-energy-scale spectra [*V*_b_ = 500 mV, *I*_t_ = 100 pA, *V*_mod_ = 3.54 mV (rms)] of the MTB in different states. The spectra have been offset vertically for clarity. The inset figure shows a narrow energy range of a characteristic band onset, which measures at 270 and 290 meV for the zero-energy state and the in-phase state, respectively. (e) MTB calculated bands. The red (blue) horizontal line depicts *E_F_* with (without) charged electrons. The color bar depicts the spectral weight of the state density on the MTB. Black arrows mark the edge states of the constructed simulation structure. The *X* point corresponds to 0.95 Å^−1^. (f) Optimized atomic structure of the MTB with charged electrons. The lattice relaxation from charging are indicated with red arrows. (g) Calculated differential charge distribution of the charged relative to the uncharged MTB. The positive (negative) charge-density difference is represented by yellow (blue) areas. The isosurface value is 1 × 10^−3^ electrons/Å^3^.

These observations suggest that the low-energy states and the QWSs are implicitly related and are thereafter treated as discrete levels by scrutinizing their node numbers. In Type-1 MTBs, the node number differs by one for adjacent levels (Fig. 3a). In Type-2 MTBs, similar node-increment rules are applied except that the two low-energy levels have the same node number (Fig. 3c). This implies that (i) the low-energy levels may also be QWSs and (ii) the two in-phase levels could be essentially from the same QWS, which is gapped by spin degeneracy lifting via a Mott-like insulating mechanism, as in [19]; (iii) the Coulomb interaction should also exist in Type-1 MTBs, which evokes an out-of-phase gap.

These implications can be examined by adjusting the electron filling, for which we expect an effect of the Coulomb interaction. By applying tip pulses of 1.9 V, all discrete levels of an out-of-phase MTB (Fig. 3a) rigidly shift toward higher energy, suggesting that the pulses change the residual charge in the MTB. The out-of-phase gap disappears immediately when the upper gap edge level partially overlaps with *E_F_* (Fig. 3b). In this new state, dubbed the zero-energy state, all the discrete levels become equidistant in energy with the adjacent level-node differing by one. The zero-energy state is irrelevant to the topological end state [24] because it is distributed throughout the MTB. Further pulsing drives the MTB into an in-phase state (Fig. 3c). The zero-energy state is unstable and can spontaneously transform into an in-phase or out-of-phase state during the spectroscopic measurements. Such phase transitions are fully reversible with opposite polarity pulses of −1.9 V (Note 3 in the Supplementary Material).

The different states of the MTBs have the same overall electronic structure (Fig. 3d). *E_F_* of the MTB is tuned by pulses, demonstrating that it accommodates additional charges, as is evident from the energy shift of the characteristic band onset (Fig. 3d, inset). Because the residual charge in the MTB is changed by the pulses, the center of the low-energy gap changes correspondingly. The additional charge is not trapped by the localized defects below the graphene [25], which would otherwise cause electronic inhomogeneity along the MTB. To understand the charge stabilization mechanism, DFT calculations were performed, with one additional electron added per six MTB units. This results in a uniform increase in *E_F_* by 81 meV (Fig. 3e). Concomitantly, the MTB Se–Se (Mo–Mo) bond length slightly increases by 0.024 pm (0.0073 pm) (Fig. 3f) and the added charges are distributed mainly on the Mo atoms of the MTB (Fig. 3g) as well as on the edges of the simulation structure (Note 4 in the Supplementary Material). Hence, the collective structural relaxation of the MTB bonds stabilizes the injected charge through a polaronic process [26].

Hereinafter, we elucidate the ground states of the MTBs. The Peierls-type CDW state suggested in [18] could be excluded because CDW should be anti-phase, contradicting our in-phase state, and the CDW gap should be invariant with the wire length and electron filling, which is distinct from Figs 2c and d and 3a–c. Notably, in the 2D case, in contrast to the 1D case, the anti-phase relation of the CDW state could be violated in specific systems by other factors, such as the special band structure [27] or strong lattice distortions [28]. The MTB can be considered an elongated quantum dot whose capacitance is proportional to *L*. This may generate a classical CB gap that is sensitive to electron filling [29]. However, this is unlikely. First, the capacitance of a monolayer MTB of a given length relative to the substrate should be approximately twice that of a bilayer MTB, which contradicts Fig. 2c and d. Second, the capacitance of the MTBs on HOPG should differ from that on graphene, but their spectral gaps exhibit the same length dependence (Fig. 2c–f). Furthermore, the spectral gap sizes are invariant upon changing the tip–MTB separations, whereas the capacitance varies (Note 5 in the Supplementary Material). Third, tip-pulsing a half-covered MTB and two contacting MTBs can only switch the ground state of the partial MTB while leaving the other half intact (Note 5 in the Supplementary Material). The long-range Coulomb interactions would mutually affect the ground states of the two contacting MTBs. This suggests that the Coulomb interaction is essentially local and drives the MTB into correlated insulating states.

We discuss the nature of the different correlated states of the MTB. In the case of the zero-energy state, the tail of the zero-energy discrete levels overlaps the Fermi level of the substrate (see Supplementary Fig. S9), which allows the charge density to fluctuate between the MTB and substrate without an energy cost. The charge fluctuation can screen the Coulomb interaction, rendering the intrinsic QWS energies detected. It is observed that, at the zero-energy state, the center of the zero-energy discrete level is always either slightly above or below *E*_F_ (Supplementary Fig. S9). With further pulsing, the center of the discrete level shifts right at *E*_F_ and the *intra*-level Coulomb repulsion opens the correlated spin–split gap, i.e. the in-phase state. Because the spin–split gap comes from the same discrete level, its spatial charge order is maintained in phase. For scenarios in which *E*_F_ is located between the two QWS levels, i.e. the out-of-phase state, the *inter*-level Coulomb interaction contributes an additional gap opening to the otherwise equidistantly spaced QWS energies. As the two discrete levels around *E*_F_ are two QWSs with adjacent wave vectors, their real-space charge-density oscillations are out of phase in the middle of the MTB, whose phase difference gradually diminishes when approaching the wire ends.

To quantitatively depict such an ascription, we consider a 1D Hubbard model [30] in a finite chain with *N* atoms to describe the MTB, which is determined as follows:


(1) \begin{eqnarray*} H\ &=& \ - \mathop \sum \limits_{i,\sigma } [t(c_{i,\sigma }^\dagger {c}_{i + 1,\sigma } + c_{i + 1,\sigma }^\dagger {c}_{i,\sigma }) \\ && - \mu {n}_{i,\sigma }] + \ \frac{U}{2}\mathop \sum \limits_i {({n}_i)}^2, \end{eqnarray*}

where the notations are standards. Confinement of the first term in Equation (1) on the finite chain results in discrete QWSs, and their eigen functions can be labeled by wave vector *k*.

**Figure 4.**
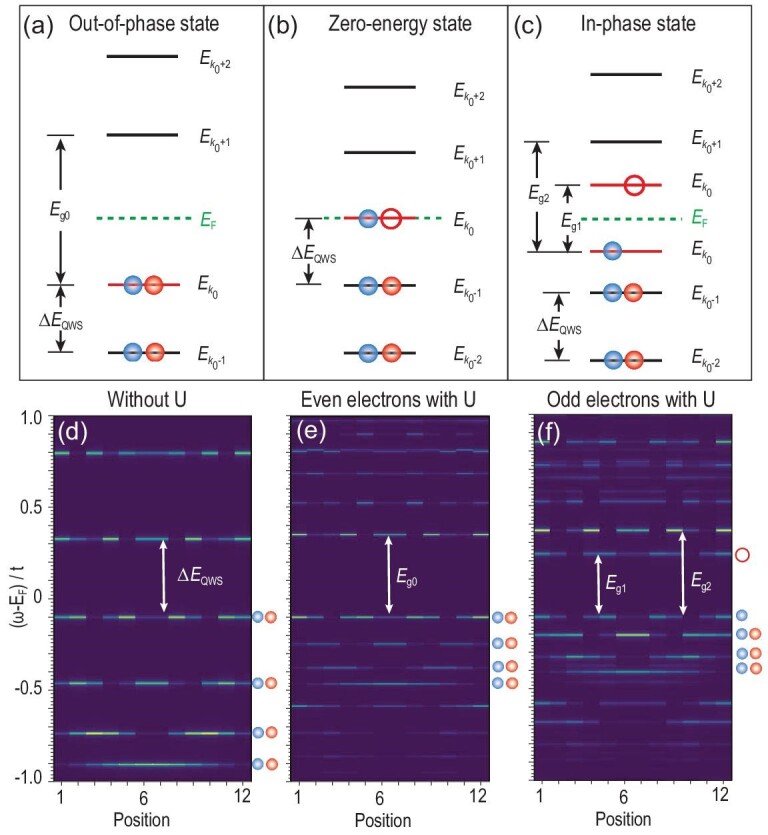
Results of Hubbard model on a finite chain. (a)–(c) Schematics showing an energy-level diagram at the mean-field level, i.e. (a) out-of-phase state, (b) zero-energy state and (c) in-phase state, respectively. Each level is marked with its wave vector and the *E_k_*_0_ level is highlighted in red. (d)–(f) Intensity plot of the LDOS on a 12-site chain with an open-boundary condition obtained by exact diagonalization method with (d) *U* = 0, eight electrons, (e) *U* = 8.0*t*, eight electrons and (f) *U* = 8.0*t*, seven electrons. The Type-1 and Type-2 MTBs have an even and an odd number of electrons, respectively. The spin-up (spin-down) electrons are depicted with red (blue) balls. The solid (hollow) balls represent electrons residing in occupied (unoccupied) levels.

First, we perform a mean-field analysis, where the Fock term in the Hubbard interaction can be neglected [31], and the effective Coulomb interaction is as follows:


(2) \begin{eqnarray*} {H}_U = \mathop \sum \limits_k {u}_1{n}_{k, \uparrow }{n}_{k, \downarrow } + \frac{1}{2}\mathop \sum \limits_{k \ne k^{\prime}} {u}_2{n}_k{n}_{k^{\prime}},\ \end{eqnarray*}

where ${u}_1 = \frac{{3U}}{{2N}}\ $and ${u}_2 = \frac{U}{N}\ $. At low energies, only the Coulomb interaction between the two relevant one-electron states, namely the highest occupied level ${k}_0$and an unoccupied level ${k}_t$hosting the tunneling electron, must be considered. In the case of the doubly occupied ${k}_0$state, corresponding to the Type-1 MTB, electron tunneling into any ${k}_t$state experiences the Coulomb interaction of two electrons, leading to a spectral gap *E_g_*_0_ = Δ*E*_QWS_ + 2*u*_2_ (Fig. 4a). Similarly, when the ${k}_0$ state is singly occupied, corresponding to the Type-2 MTB, electron tunneling into any ${k}_t$ state only experiences the Coulomb interaction of one electron whose strength depends on the specific ${k}_t$ state. Then, the spectral gap would be *E_g_*_1_ = *u*_1_ for tunneling into the lowest unoccupied level (*k_t_* = *k*_0_) and *E_g_*_2_ = Δ*E*_QWS_ + *u*_2_ for tunneling into the second lowest unoccupied level (${k}_t$ ≠ ${k}_0$) (Fig. 4c). In contrast, if the ${k}_0$state is at *E_F_*, charge fluctuations effectively screen the Coulomb interaction, removing the correlated gap (Fig. 4b). Such modeling analysis can determine *U* using experimentally extracted parameters (Note 7 in the Supplementary Material). For the in-phase state, a comparison of the experimental and theoretical *E_g_*_1_ yields *U* = 1.9 ± 0.1 eV. A similar comparison of the out-of-phase state determines a similar *U* = 1.4 ± 0.2 eV. The theory also yields $\frac{{{E}_{g2}}}{{{E}_{g1}}}$ = 1.43 and $\frac{{{E}_{g0}}}{{\Delta {E}_{QWS}}} = 2.22$, conforming to the measured respective values of 1.68 ± 0.07 and 2.25 ± 0.14.

The picture of the in-phase and out-of-phase states can be numerically verified beyond the mean-field level. In Fig. 4d–f, the intensity plot of the local density of states (LDOS) of Equation (1) in a 12-site chain with an open-boundary condition obtained using the exact diagonalization method is shown. Figure 4d presents the result of the *U* = 0 case, exhibiting discrete QWSs with different numbers of nodes. Owing to the finite-size effect, the gap between different QWSs varies, and that relevant to Fig. 4e and f is $\Delta {E}_{QWS}\sim0.43t$. In Fig. 4e and f, a strong Hubbard interaction (*U* = 8.0*t*) is turned on for even-filled (eight electrons) and odd-filled (seven electrons) systems, respectively. When the system is even-filled (Fig. 4e), the node number of the LDOS across *E_F_* changes by one, and the spectral gap is ${E}_{g0}\sim0.45t$, which is slightly greater than $\Delta {E}_{QWS}$. In contrast, when the system is odd-filled (Fig. 4f), the node number of the LDOS remains the same, with the first spectral gap ${E}_{g1}\sim0.34t$ and the second ${E}_{g2}\sim0.47t$. This numerical result reproduces the observed in-phase state (corresponding to Fig. 4f) and out-of-phase state (corresponding to Fig. 4e) well, except for the different gap ratios $\frac{{{E}_{g2}}}{{{E}_{g1}}}\sim1.38$ and $\frac{{{E}_{g0}}}{{\Delta {E}_{QWS}}}\sim1.05$. Such deviations could be attributed to finite-size effects, as the realized systems are much larger in the experiments. However, it should be noted that the node-change feature across *E_F_* is robust against the system size, electron numbers and even the Hubbard interaction strength, as long as the electron number parity is fixed (Note 8 in the Supplementary Material). Moreover, as indicated by the colors of the balls in Fig. 4d–f, we observe that in the out-of-phase state, the two energy levels near *E_F_* always contain contributions from both spin-up and spin-down electrons, whereas in the in-phase state, the two energy levels near *E_F_* with the same number of nodes always come from electrons with opposite spins, which is consistent with the mean-field picture.

As expressed in Equation (2), the electron confinement of the wave functions renders the effective Coulomb interaction normalized by the chain site *N*, which becomes the on-site *U* at the single-site limit. The effective Coulomb energy is analogous to the charging energy *e*^2^*/*(2*C*) in the classical CB effect, where the capacitance *C* is proportional to the wire length. Thus, its dictation on the correlated insulating state can be considered a Hubbard-type CB effect. The on-site *U* suggests TLL behavior in the MTB [22,23], as observed from the Fourier transformation of a conductance plot along the MTB, exhibiting spin-charge separated dispersions (Note 9 in the Supplementary Material).

## CONCLUSION

In conclusion, we discovered a Hubbard-type CB effect, which could establish an alternative platform for emulating the Hubbard model in artificial quantum systems, as recently demonstrated with bosons [32] or fermions [33]. The Hubbard-type CB effect does not explicitly require inputs from the specific system of MoSe_2_ MTB, which is beyond the scope of clarifying the controversies over its ground state. Such an effect, in contrast to the classical CB effect due to the static Coulomb repulsion energy of electrons, features a quantum many-body effect due to the strong interactions between electrons. Our strategy of *in situ* electron-filling adjustment allows the addressing and manipulation of individual nano-objects with atomic-scale precision, which may foster the study of correlation physics.

## MATERIALS AND METHODS

The experiments were conducted using custom-made Unisoku cryogenic STM [34] at 4 K. An electrochemically etched W wire was used as the STM tip, which was cleaned on an Ag(111) surface prior to performing the measurements. Tunneling spectra were obtained using a lock-in technique with a modulation voltage of 983 Hz. Monolayer and bilayer MoSe_2_ films were grown on graphene-covered SiC(0001) substrates or HOPG substrates with molecular beam epitaxy by co-depositing high-purity Se (purity 99.999%) and Mo (purity 99.95%) atoms with a flux ratio of ∼10:1. The substrate temperature was maintained at ∼530 K during the growth, with subsequent annealing at 870 K for 10 mins. The detailed preparation procedures for the graphene substrate are described in [5]. The HOPG substrate was cleaved *ex situ* and further degassed in a vacuum chamber at ∼1170 K for 0.5–2 h before MoSe_2_ growth.

DFT calculations were performed using the Vienna *ab initio* simulation package (VASP) [35] with the Perdew–Burke–Ernzerhof (PBE) exchange-correlation functional [36] and projector-augmented wave pseudo-potential. A plane-wave basis set with an energy cut-off of 300 eV was applied. We used periodic boundary conditions along the MTB and added a vacuum layer of 10 Å on each side of the structure perpendicular to the MTB. In addition, a vacuum layer of 15 Å was placed normal to the basal plane, which was sufficiently large to eliminate artificial interactions among the periodic images. The structure model contained 34 Se atoms and 16 Mo atoms with a lattice constant of 3.31 Å along the MTB. Geometry optimization was performed until the residual force on each atom was <0.02 eV/Å. We used a 1 × 18 × 1 k-mesh for structure relaxation and electronic structure calculations, and 51 k-point sampling along the MTB for the band structure calculation.

## Supplementary Material

nwac210_Supplemental_File
