# Supplementary material for: Manipulating Hubbard-type Coulomb blockade effect of metallic wires embedded in an insulator
Source: Natl Sci Rev. 2022 Oct 4;10(3):nwac210. doi: 10.1093/nsr/nwac210 (PMC10081919; doi:10.1093/nsr/nwac210)
Supplement: nwac210_Supplemental_File [file nwac210_supplemental_file.docx]

**Supplementary Information for**

**Manipulating Hubbard-type Coulomb blockade effect of metallic wires embedded in an insulator**

Xing Yang^1^, Zhao-Long Gu^2^, Huimin Wang^3^, Jing-Jing Xian^1^, Sheng Meng^3^, Naoto Nagaosa^4,5^, Wen-Hao Zhang^1^, Hai-Wen Liu^6^, Zi-Heng Ling^1^, Kai Fan^1^, Zhi-Mo Zhang^1^, Le Qin^1^, Zhi-Hao Zhang^1^, Yan Liang^1^, Jian-Xin Li^2,7^,Ying-Shuang Fu^1^

^1^School of Physics and Wuhan National High Magnetic Field Center, Huazhong University of Science and Technology, Wuhan 430074, China

^2^National Laboratory of Solid State Microstructures and Department of Physics, Nanjing University, Nanjing 210093, China

^3^Beijing National Laboratory for Condensed Matter Physics and Institute of Physics, Chinese Academy of Sciences, Beijing 100190, China

^4^RIKEN Center for Emergent Matter Science (CEMS), Wako, Saitama 351-0198, Japan

^5^Department of Applied Physics, University of Tokyo, Tokyo 113-8656, Japan

^6^Center for Advanced Quantum Studies, Department of Physics, Beijing Normal University, Beijing 100875, China

^7^Collaborative Innovation Center of Advanced Microstructures, Nanjing University, Nanjing 210093, China

**SUPPLEMENTARY NOTES**

- - - 1. **Atomic resolution imaging of MTB**

The crystal structure of the MTB, as shown in the inset of Fig. 1(b), was identified in two independent studies [S1,S2]. However, the calculated density of states (DOS) showed substantial inconsistency presumably on account of the different bond lengths defined for the MTB. In this regard, we performed atomic-resolution imaging of the MTB with a decorated tip obtained by controlled dipping into the MoSe_2_ film. The tip apex is then terminated with Se atom or MoSe_2_ piece. Once the tip apex is decorated with nonconductive species, irrespective of Se or MoSe_2_, the tip has to be close to the surface to maintain the tunneling current. In such a case, tip is sensitive to the atomic corrugations of the surface, giving rise to atomic resolutions. Otherwise, the metallic MTB has large density of states within the band gap of MoSe_2_, and is also spatially extended. This results the tip-sample distance large when imaged with a W tip, smearing out the atomic corrugations. Thus, the decorated tip enabled imaging of its top layer Se. The imaged Se atomic lattices across the MTB, as shown in Fig. S1(a–c), are consistent with the structure in Fig. 1(b); they express an offset in the adjacent domains, as indicated by the black lines in Fig. S1(a–c). Such an offset is more evident from the Moiré pattern between the MoSe_2_ and graphene [Fig. S1(a–c)], which is discontinuous at the MTB. This finding further demonstrates that the Se lattice constant at the MTB differs from that of the inside domains. This observation is consistent with our DFT calculations, where the crystal structure of the MTB also shows different Mo–Mo bond lengths compared to that of the inside domains [Fig. S1(d)].


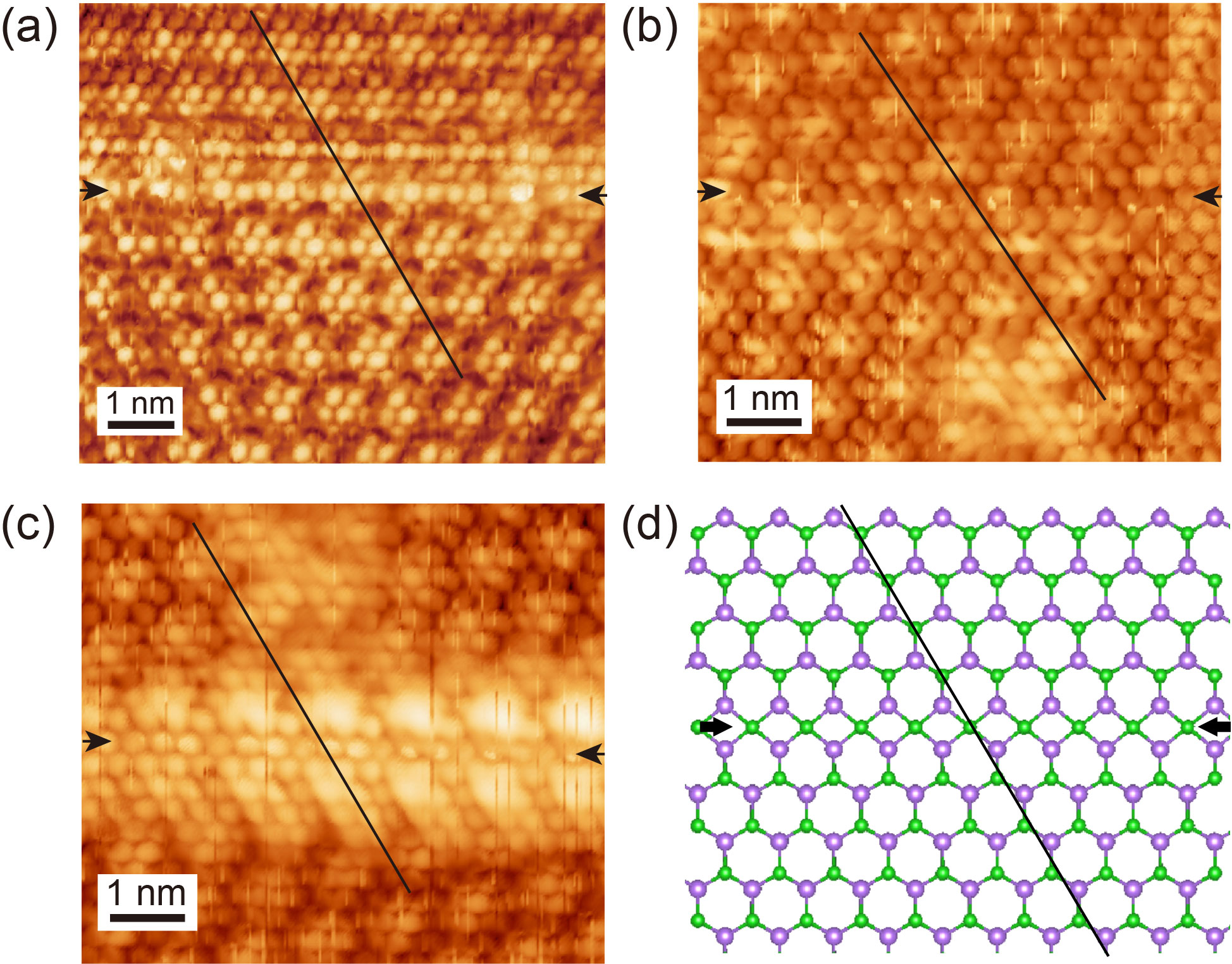


Figure S1. Atomic resolution STM image of MTB. (a–c) STM image of MTB on MoSe_2_ with different Moiré orientations relative to the graphene substrate. The images were obtained with a Se terminated tip. Imaging conditions: *V*_t_ = −10 mV, *I*_t_ = 10 pA for (a); *V*_t_ = 20 mV, *I*_t_ = 20 pA for (b); *V*_t_ = 30 mV, *I*_t_ = 20 pA for (c). (d) DFT-calculated crystal structure of an MTB. The Mo (Se) atoms are represented with purple (green) balls. For all images, black lines mark the misalignment of Se lattices of two domains adjacent to the MTB. The black arrows mark the MTB locations.

1. **Evidence of quantum well states in the MTB**

The magnified image of an individual MTB resolves the topographic modulations along the MTB (Fig. S2), implying the occurrence of charge modulations. The spatial modulation of the charge density along the MTB can be ascertained from its STM topographic images. The corrugation in the apparent height along the MTB is predominantly due to its charge density modulations since the atomic corrugation is negligibly small. Fig. S2(a) shows an STM image of an MTB at 0.4 V. Evidently, there is a topographic modulation in its apparent height along the MTB, where the modulation amplitude is larger at the two ends of the wire. This signifies that the charge modulation is due to the electron confinement effect. Under an imaging bias of 0.1 V [Fig. S2(b)], the corrugation becomes larger than that under 0.4 V. Meanwhile, Fig. S2(c) shows the line profiles obtained along the black line in (a) that was imaged under different sample biases. To compare their oscillation periods, we aligned their maximum oscillation amplitudes at the right MTB end at the vertical gray line. The corrugation period along the MTB increases with the increasing bias [Fig. S2(c)]. This demonstrates that the topographic corrugation along the MTB is due to the quantum confinement of 1D electrons by the finite length of the wire.

Further evidences supporting the quantum well states are the following. First, the discrete levels are distributed in the full spectroscopic energy range [−1.2, 0.5] eV of Fig. 1(d) of the main text. Second, their modulation periods decrease with increasing energy. Third, their energy intervals increase with a decreasing wire length (as is shown in Fig. 2).


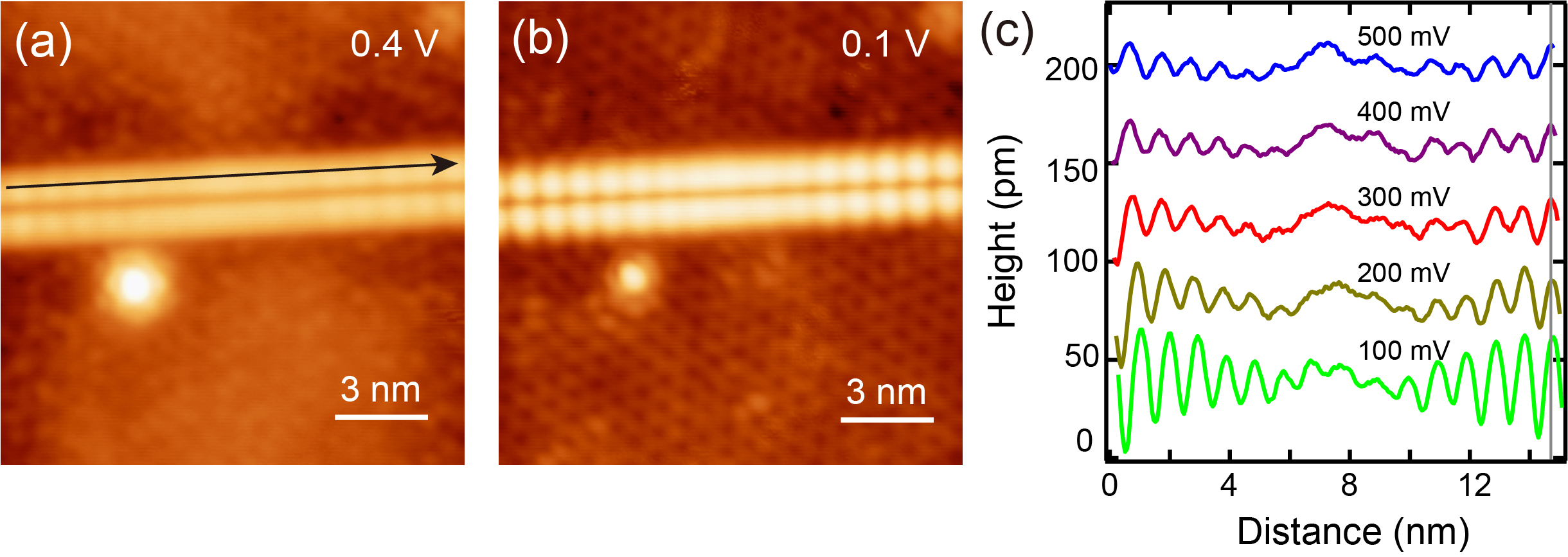


Figure S2. Topographic oscillations of an MTB at different voltages. (a,b) Typical STM images (*I*_t_ = 10 pA) of a single monolayer MTB at 0.4 and 0.1 V, respectively, showing topographic oscillations. (c) Line profiles obtained along the black arrows of (a) at different voltages. The line profiles have shifted vertically and are aligned at a grey vertical line. The topographic oscillations are more enhanced at ends of the MTB, whose period increases with an increasing bias. This demonstrates that the topographic oscillation is consistent with quantum confinement.

1. **Reversible transition of the correlated states in MTB by voltage pulses**

The correlated states of the MTB can be reversibly manipulated by the voltage pulses of the STM tip. Fig. S3depicts an example. Each spectrum in Fig. S3 is averaged from the line spectra obtained along the MTB, whose image is shown in Fig. 1(c) of the main text. Initially, the MTB is in the in-phase state (a). Its two discrete levels adjacent to *E*_F_ are the spin-split states of the same discrete level, and are thus marked with the same green triangle. After pulsing the MTB with + 1.9 V, electrons are injected into the MTB from the STM tip, shifting all the discrete levels upward in energy. Then, the MTB is transformed into the zero-energy state (b), where the discrete level marked with a green triangle is at *E*_F_ and its spin-split gap concomitantly disappears. With further pulsing of +1.9 V, the Fermi level moves between the two discrete levels marked with purple and green triangles, respectively. The Coulomb interaction subsequently drives the MTB into the out-phase state (c). With negative voltage pulsing of −1.9 V, holes are injected into the MTB, shifting all the discrete levels downward in energy. This results in a reversed transition from the out-phase state (c) to the in-phase state (e) through the zero-energy state (d). We attempted different voltage pulses and found its amplitudes from 1.2 to 2.5 V are all effective for inducing the transition.

We further evaluated the overall electronic structure of the MTB in different states in a large bias range. Fig. S4(a) and (b) show the line spectra of the same MTB at the zero-energy and in-phase states, respectively. Evidently, the overall electronic structure of the MTB does not change. In addition, the electronic states along the MTB are quite uniform except at the wire ends.


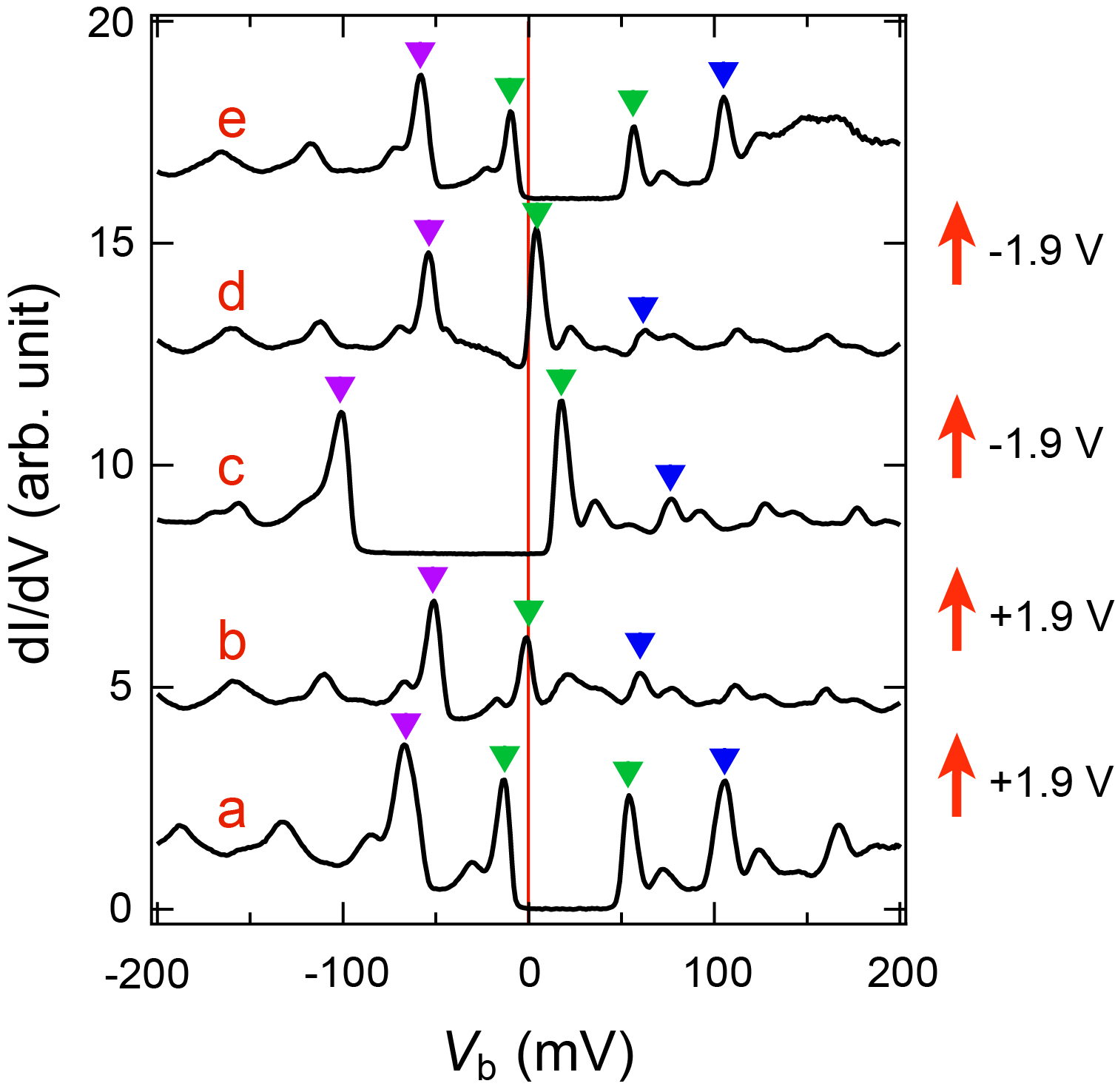


Figure S3. Reversible correlated phase transition of MTB with voltage pulses. Tunneling spectra of a monolayer MTB, which is the same as that in Fig. 3, showing reversible phase transition among different states with series of STM voltage pulses, as indicated in the figure. The spectra are offset vertically for clarity. Low-energy discrete levels close to *E*_F_ are marked with colored triangles, where the same colored triangle represents the same discrete level at different states. Each state is marked with a red letter. Spectroscopic conditions: *V*_t_ = 200 mV, *I*_t_ = 100 pA, *V*_mod_ = 1.414 mV (rms).


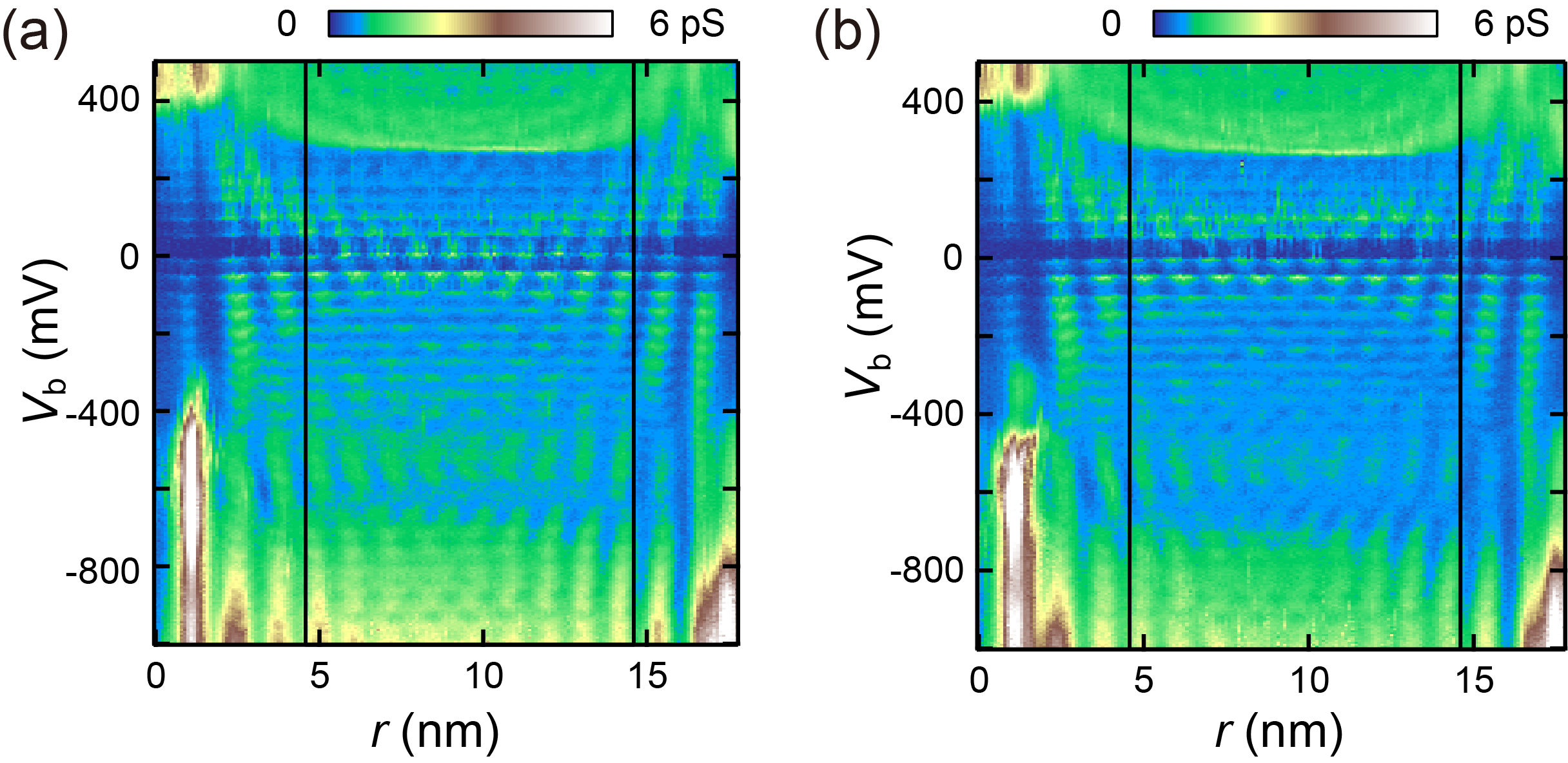


Figure S4. Conservation of overall electronic structure of MTB. (a,b) 2D conductance plot obtained along the black line in Fig. 1(c) with a large energy range showing the electronic structure of the MTB at the zero-energy state (a) and in-phase state (b), respectively. Spectroscopic conditions: *V*_t_ = 500 mV, *I*_t_ = 100 pA, *V*_mod_ = 3.54 mV (rms). The spectra in Fig. 3(d) are averaged from the spectra in (a) and (b) of the rectangle region. The overall electronic spectrum of the MTB does not change in the different states.

1. **DFT calculations on the charging of the MTB**

We provide detailed descriptions of the DFT calculations of the MTB. Fig. S5(a) shows the simulation unit cell (gray area) constructed for calculating the MTB. It consists of a monolayer MoSe2 ribbon, which is terminated with Se atoms at the edges and an MTB with a 4│4P structure MTB in the middle (indicated by the red dashed box). The calculated bands projected on the MTB are shown in Fig. 3(e) of the main text, which inevitably includes a small contribution of edge states. Subsequently, we added one additional electron per six ribbon units by changing the total number of electrons in the ribbon and we tested its response to the added charge. Fig. S5(b) shows the differential charge distribution of the charged relative to the uncharged ribbon. The added charges are mainly distributed at the Mo atoms of the MTB and the edges. We estimate that approximately 48% of the additional charges are added to the MTB.


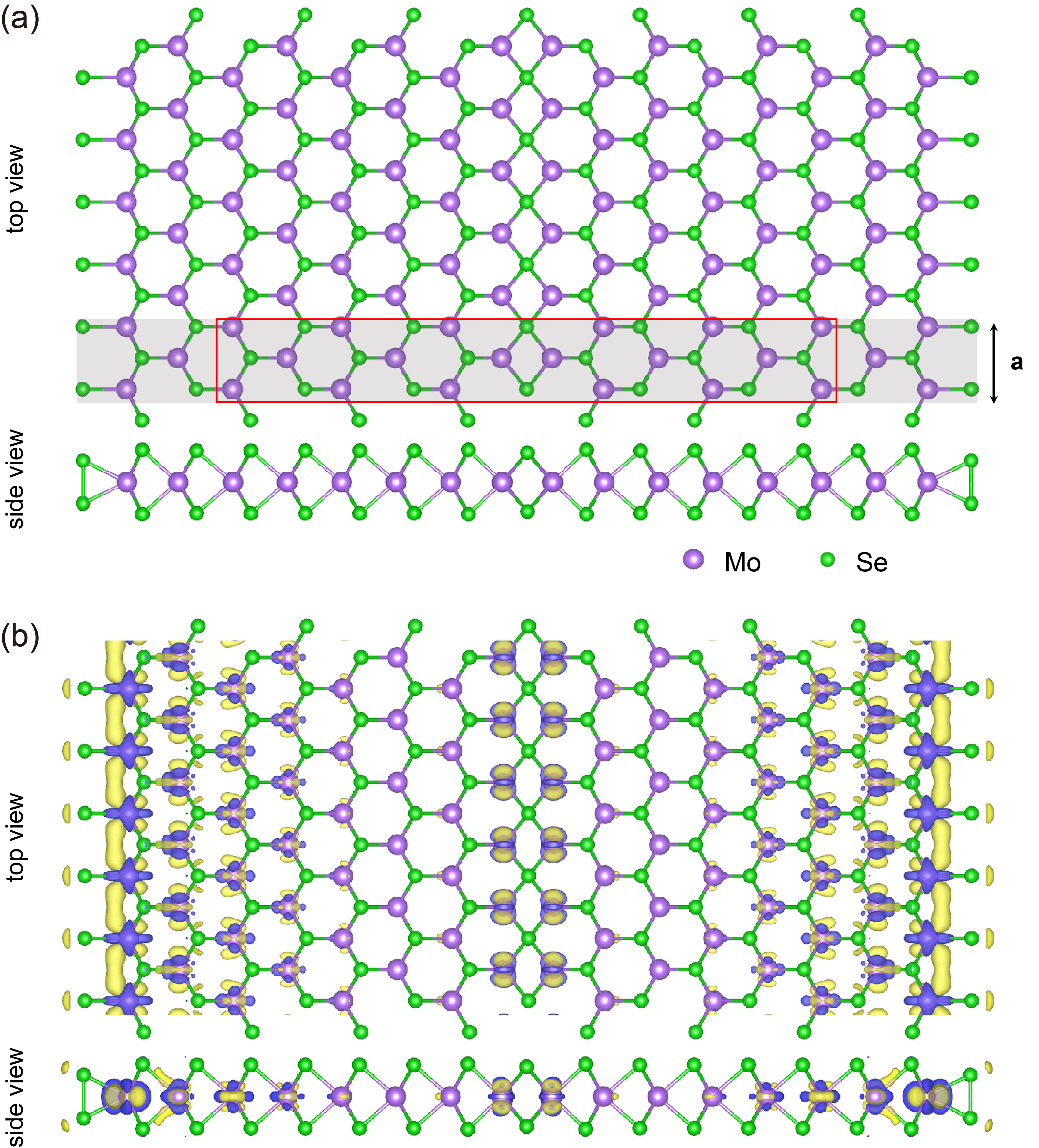


Figure S5. Constructed ribbon structure and differential charge distribution. (a) Constructed simulation unit cell (gray area) for the DFT calculation, which contains an MTB in the middle (red dashed box) and two Se-terminated edges. (b) Differential charge distribution of the ribbon after adding one additional electron per six ribbon units with respect to its uncharged state. The positive (negative) charge-density difference is represented by yellow (blue) areas. The isosurface value is 1×10^−3^ electrons/Å^3^. Note that the effective charge added to MTB is approximately 48% of that of the total charge added in the calculation owing to their redistribution to the edges of the constructed ribbon structure.

1. **Evidence of the short-range Coulomb interaction in MTB**

For the monolayer MTB supported on a HOPG substrate, a double tunneling barrier junction forms in an STM measurement configuration. The double tunneling barrier consists of the van der Waals gap at the HOPG/MTB interface and tip-MTB vacuum separation. The finite-size MTB can therefore be considered a quantum dot. If a conventional Coulomb blockade effect exists in the MTB, its Coulomb gap should be determined by its capacitance coupling to the environment, which is determined by its separation from both the tip and the HOPG substrate. To evaluate such a scenario, we measured the Coulomb gap with different tip–MTB separations, as shown in Fig. S6. Surprisingly, the Coulomb gap, marked with two gray vertical lines, does not change. This demonstrates that the Coulomb gap is of short range in nature, unlike the classical Coulomb blockade effect with long-range Coulomb interaction.

The short-range Coulomb interaction in the MTB is more rigorously evidenced from the correlated state transitions of the half-covered MTB and two contacting MTBs. Fig. S7(a) shows an STM image of a monolayer MTB, which is half-covered by the second-layer MoSe_2_. Line spectra along the MTB show that the entire wire is in the in-phase state [Fig. S7(b)]. There is a band offset at the edge of the second layer of MoSe_2_, which is caused by an electrostatic potential exerted on the MTB from the covered dielectric second layer MoSe_2_.Interestingly, after pulsing the uncovered half of the MTB, it changes to the out-phase state [Fig. S7(c)]. However, the covered half is maintained in the in-phase state. This demonstrates that the Coulomb interaction in the MTB is very local. Otherwise, if the Coulomb interaction is in the long range, the entire MTB should be in the same state. Similar phenomena are observed in two contacting monolayer MTBs, whose image is shown in Fig. S8(a). Line spectra along the two contacting MTBs indicate that the upper and lower MTBs are in the in-phase and out-phase states, respectively [Fig. S8(b)]. Voltage pulses are applied to the lower MTB, which induces a phase transition to the zero-energy state. Meanwhile, the upper MTB remains in the out-phase state [Fig. S8(c)]. This finding serves as additional supporting evidence for the local nature of the Coulomb interaction in the MTB.


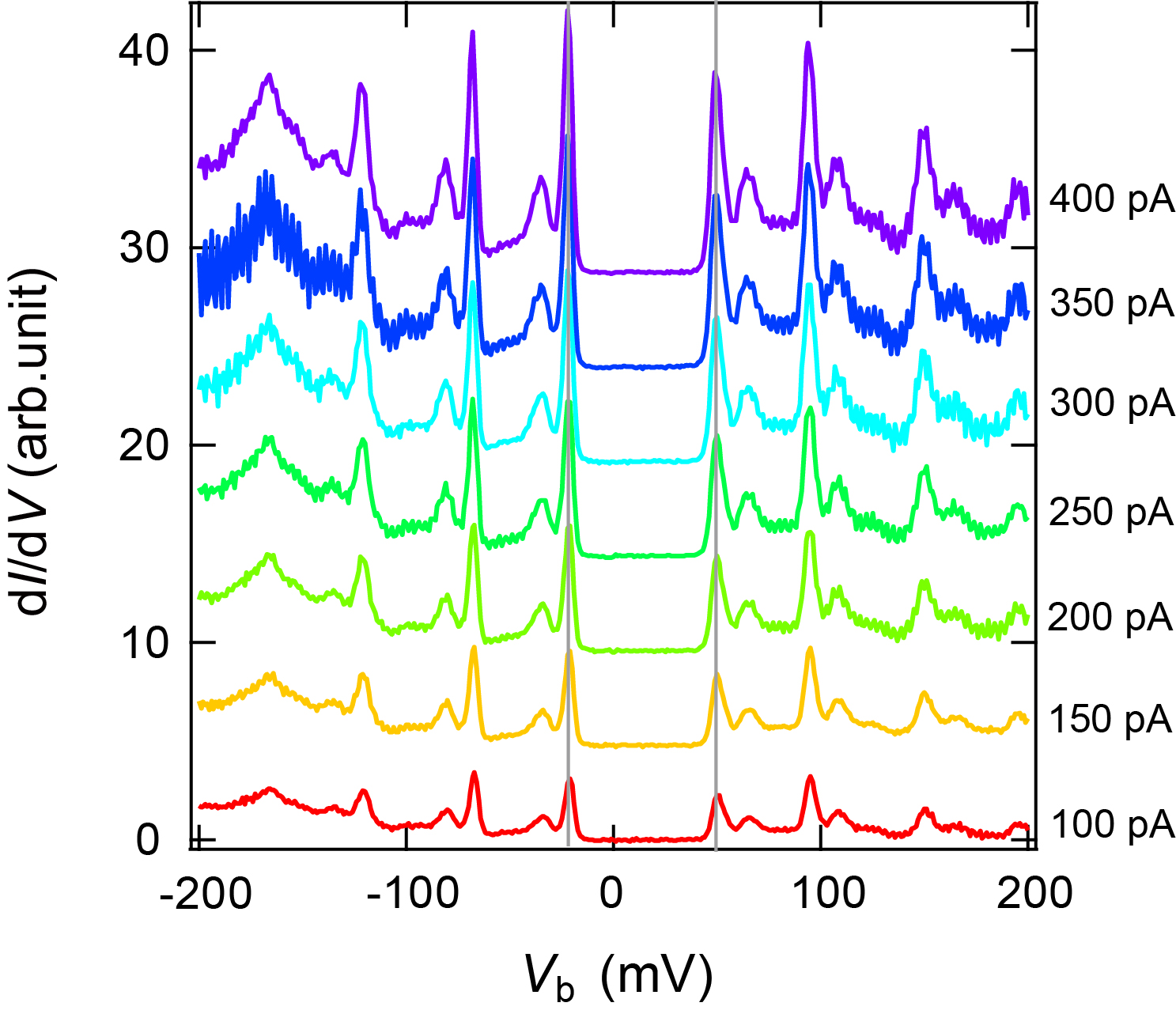


Figure S6. Tunneling spectra of a monolayer MTB on HOPG substrate with different tip-sample separations. The spectra have been offset vertically for clarity. Two vertical grey lines mark the peak energies of low-energy states of the MTB. The peak energies for each spectrum are constant against changing tip-sample separations. Spectroscopic conditions: *V*_t_ = 200 mV, *V*_mod_=1.414 mV (rms). The set currents have been marked for each spectrum.


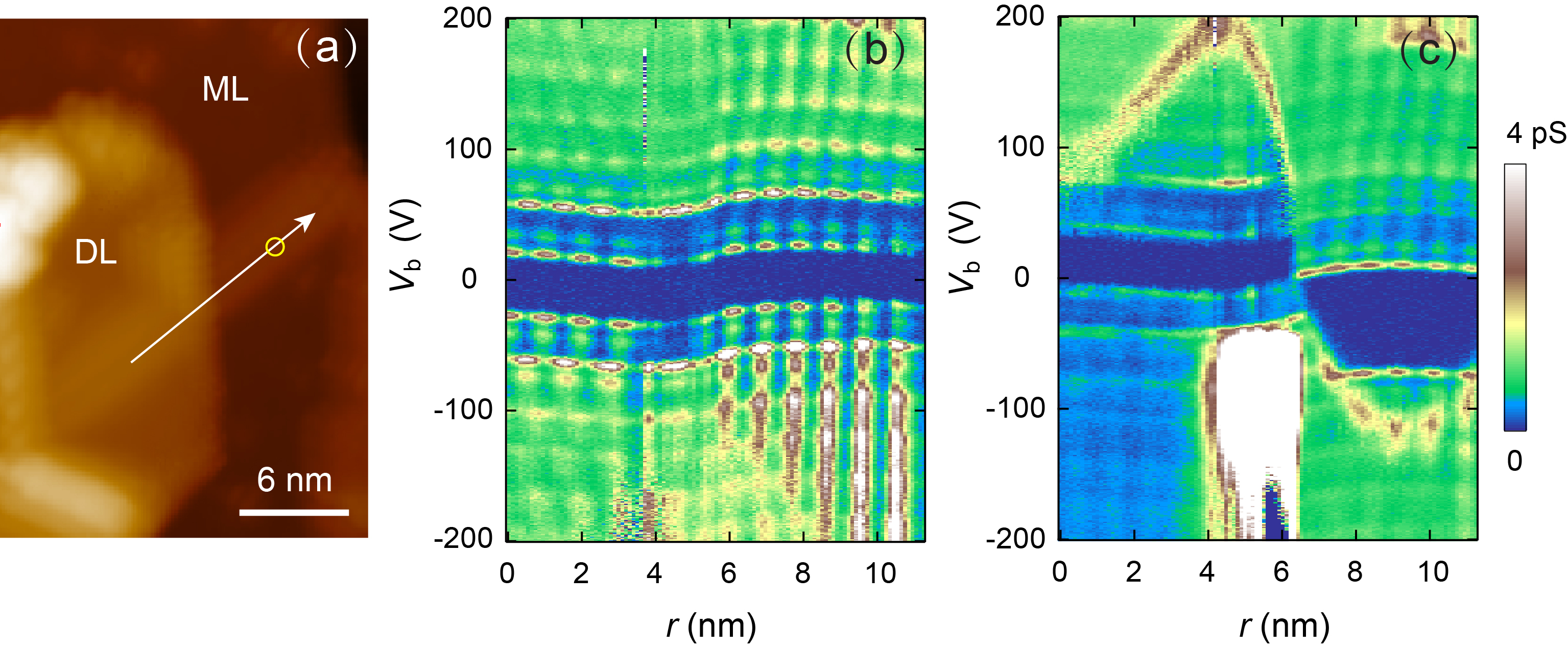


Figure S7. Tunneling spectra of a half-covered MTB. (a) STM image (*V*_t_ = 500 mV, *I*_t_ = 20 pA) of a monolayer (ML) MTB half-covered by double layer (DL) MoSe_2_. (b,c) Tunneling spectra [*V*_t_ = 200 mV, *I*_t_ = 100 pA, *V*_mod_= 1.414 mV (rms)] of the half-covered MTB obtained along the white line in (a) before (b) and after (c) the application of voltage pulses, respectively. The yellow circle marks the location of the applied pulses.


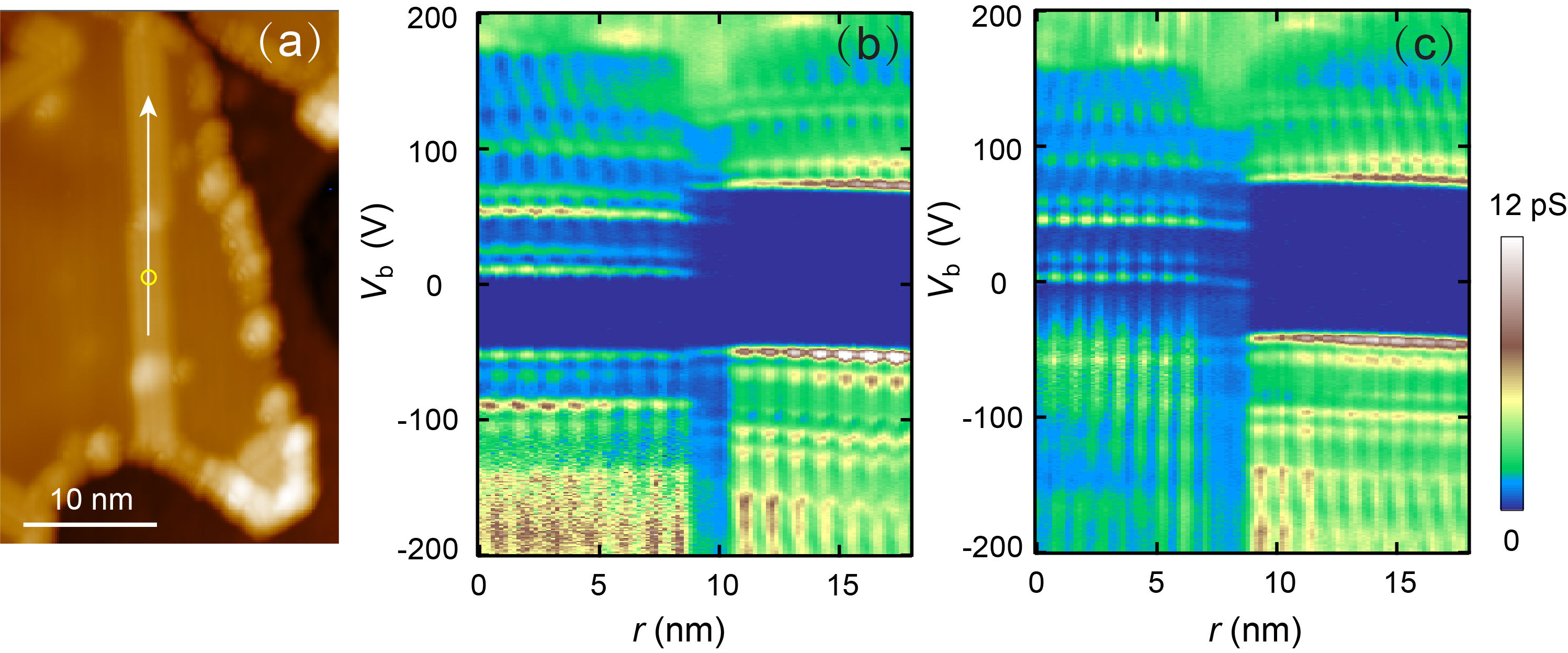
Figure S8. Tunneling spectra of two adjacent MTBs. (a) STM image (*V*_t_ = 500 mV, *I*_t_ = 10 pA) of two contacting monolayer MTBs that are laterally shifted toward each other by a lattice constant. (b,c) Tunneling spectra [*V*_t_ = 200 mV, *I*_t_ = 200 pA, *V*_mod_ = 1.414 mV (rms)] of the two MTBs obtained along the white line in (a) before (b) and after (c) application of voltage pulses, respectively. The yellow circle marks the location of the applied pulses.

1. **Features of the zero-energy state**


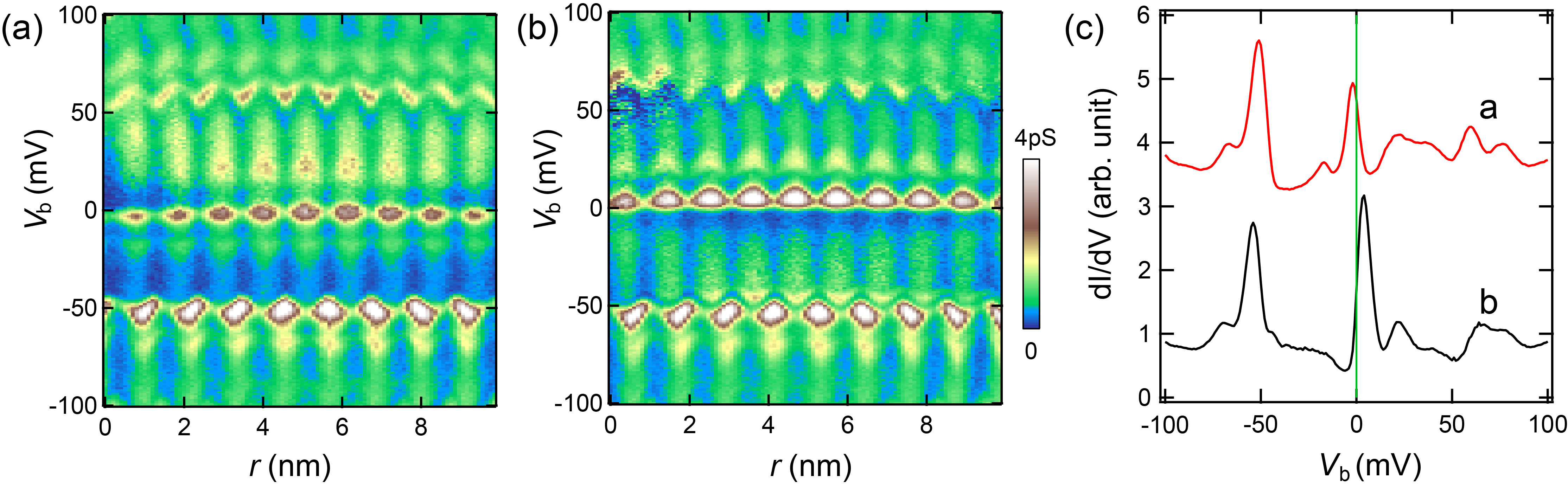


Figure S9. Zero-energy state of MTB. (a,b) 2D conductance plot [*V*_t_ = 200 mV, *I*_t_ = 100 pA, *V*_mod_ = 1.414 mV (rms)] of the same MTB, whose STM image is shown in Fig. 1(c), showing its zero-energy state slightly below (a) and above (b) *E*_F_, respectively. (c) Averaged spectra of (a) and (b). The Fermi level is marked with a green line. The spectra have been offset vertically for clarity.

1. **Statistics on the energy spacing between quantum well states with MTB length**

Due to the confinement by the finite length of the MTB, electrons are confined into quantized quantum well states (QWSs). Around the narrow energy range around the Fermi level, the energy dispersion of the MTB can be considered as linear. This approximation predicts the QWSs are equally spaced in energy, whose energy spacing ($\Delta E_{QWS}$) is inversely proportional to the MTB length (*L*) with a proportional factor $E_{Q}$. Thus, we have $\Delta E_{QWS}=\frac{E_{Q}}{L}$. Fig. S10 shows the statistics of the measured $\Delta E_{QWS}$ with respect to *L*. Note that we didn’t differentiate the monolayer and bilayer MTB. It can be seen from Fig. S10 that the two types of MTBs on both graphene and HOPG substrates have their $\Delta E_{QWS}$ decreases monotonically with increasing *L*. By fitting the data on graphene, we can determine a *E*_Q_ of 0.74±0.02 eV·nm for the type-1 MTB, namely the MTB in the out-of-phase state. Similar fitting to the type-2 MTB (in-phase state) on graphene gives a *E*_Q_ of 0.73±0.02 eV·nm. Those two values are similar, demonstrating the QWSs spacing is irrelevant to the ground state of the MTB. The *E*_Q_ value can also be estimated from the dispersion of the DFT calculation. The energy of the *n*-th QWS is . By fitting the MTB band of Fig. 3(e) within the energy range of [-150 meV, +150 meV] with a linear dispersion, we have = 0.248 eV·nm. This yields *E*_Q_ =0.77 eV·nm, which is also consistent with the experiment. With such experimentally extracted expression of $\Delta E_{QWS}$, the Hubbard interaction strength U could be determined by comparing the experimental and theoretical expression of $E_{g0}$or $E_{g1}$, as has been stated in the main text.


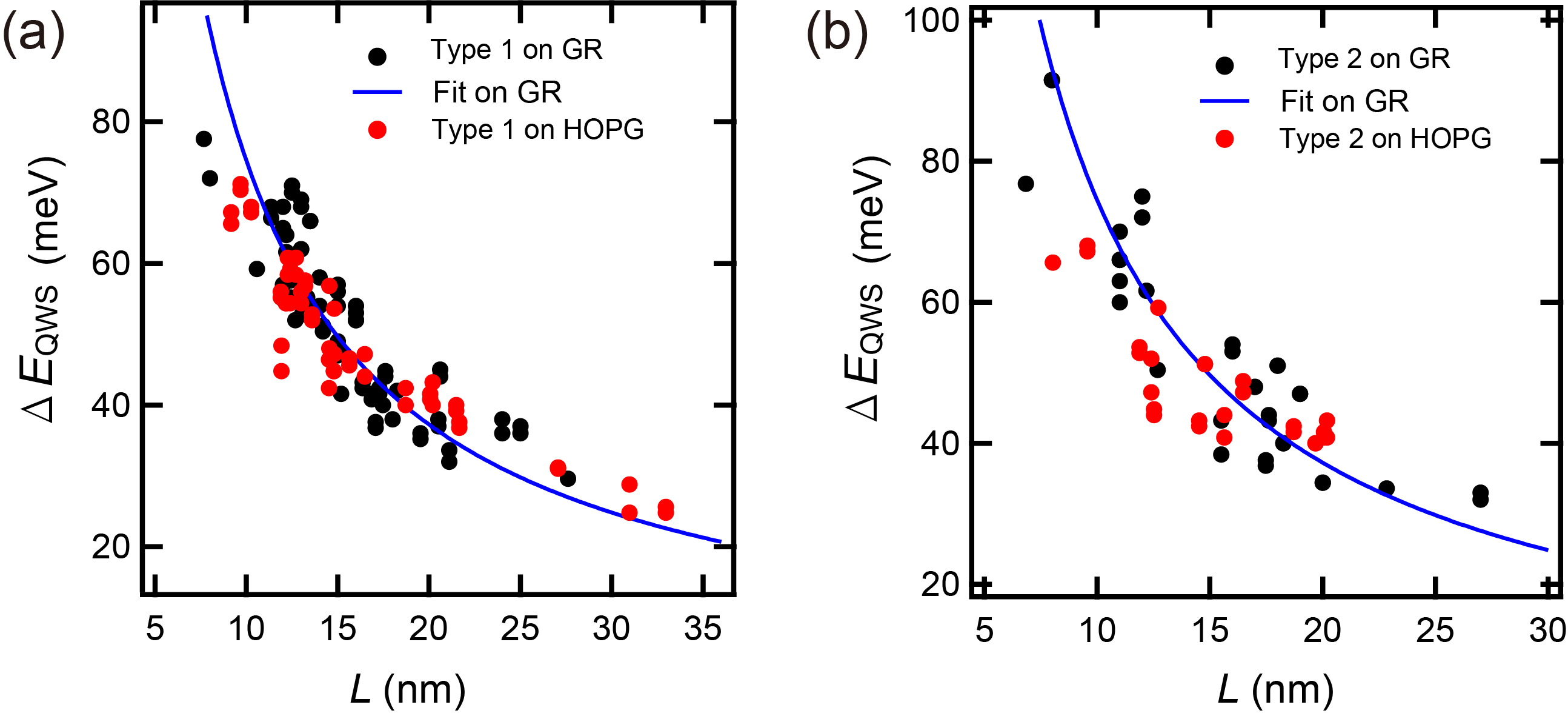


Figure S10. Statistics of *E*_Q_. (a) Statistics of the $\Delta E_{QWS}$ to *L* for the type 1 MTB on graphene (black dots) and HOPG (red dots). The blue curve is a fitting to the data on graphene (GR) with an inverse= proportional relation, which gives *E*_Q_ = 0.74±0.02 eV·nm. (b) Similar as (a), but for the type 2 MTB. The fitting gives *E*_Q_ = 0.73±0.02 eV·nm.

**8．Robustness of the node change feature across the Fermi energy in ED simulations**

The one-dimensional Hubbard model is given by,

$$H=\sum_{i\sigma} [-t（c_{i,\sigma}^{+}c_{i+1,\sigma}+h.c.)-\mu n_{i\sigma}]+\frac{U}{2}\sum_{i} {n_{i}}^{2},$$

where the notations are standard. We solve this model in a finite-size chain with the open boundary condition by the exact diagonalization (ED) method. The results are presented in Fig.4 (d)-(f) of the main text with 12-site chain. In Fig. S11, another set of results are presented with a different choice of the system size, electron filling and Hubbard interaction strength. Apparently, the same node change feature across the Fermi energy could be observed although a different set of gap values are obtained $\Delta E_{QWS}\simeq0.22t$, $E_{g0}\simeq E_{g2}\simeq0.25t$, and$E_{g1}\simeq0.17t$. In general, such node change feature is robust against the system size, electron numbers and the Hubbard interaction strength as long as the electron number parity in the correlated one-dimensional system is fixed.


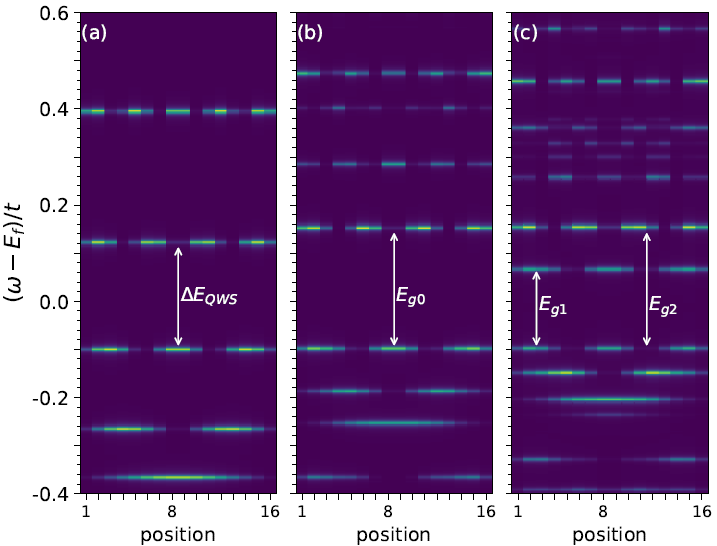


Figure S11. Intensity plot of the local density of states of the one-dimensional Hubbard model with 16 sites subject to open boundary condition: (a) U=0, *N*_e_=6, (b) U=4.0t, *N*_e_=6, and (c) U=4.0t, *N*_e_=5.

1. **Spin-charge separation in 1D metallic states of MTB**

For 1D metallic states, electron–electron interactions drive the 1D conductor into a Tomonaga–Luttinger liquid (TTL) state. A hallmark of the TLL state is spin-charge separation in its low-energy excitation, which splits the original single branch band with linear dispersion into two branches for spin and charge, respectively. When the 1D metal with TLL state is subject to a finite size confinement effect, standing waves occur in a manner similar to that of the Fermi liquid. However, the fast Fourier transformation of the standing waves yields two branches, which correspond to the spin and charge branch, respectively. This strategy of identifying the TLL behavior was reported in the MTB of the related compound MoS_2_ in Ref. 21 of the main text. Here, we adopted a similar analysis scheme for our MTB. Fig. S12(a) shows a 2D conductance plot obtained along an MTB in the in-phase state. The node numbers for each discrete level are marked. The Fourier transformation [Fig. S12(b)] indicates two branches below approximately −10 meV, which highlights the TLL behavior. However, its counterpart branches at the positive bias are not as clear as those at the negative bias.  It is noted that phonon replica of the 14 meV mode should exist for both the spin and charge branches. Nevertheless, since the separation of the spin and charge branches is not large, we didn’t explicitly mention it in our model and the schematic drawings of Fig. 4.


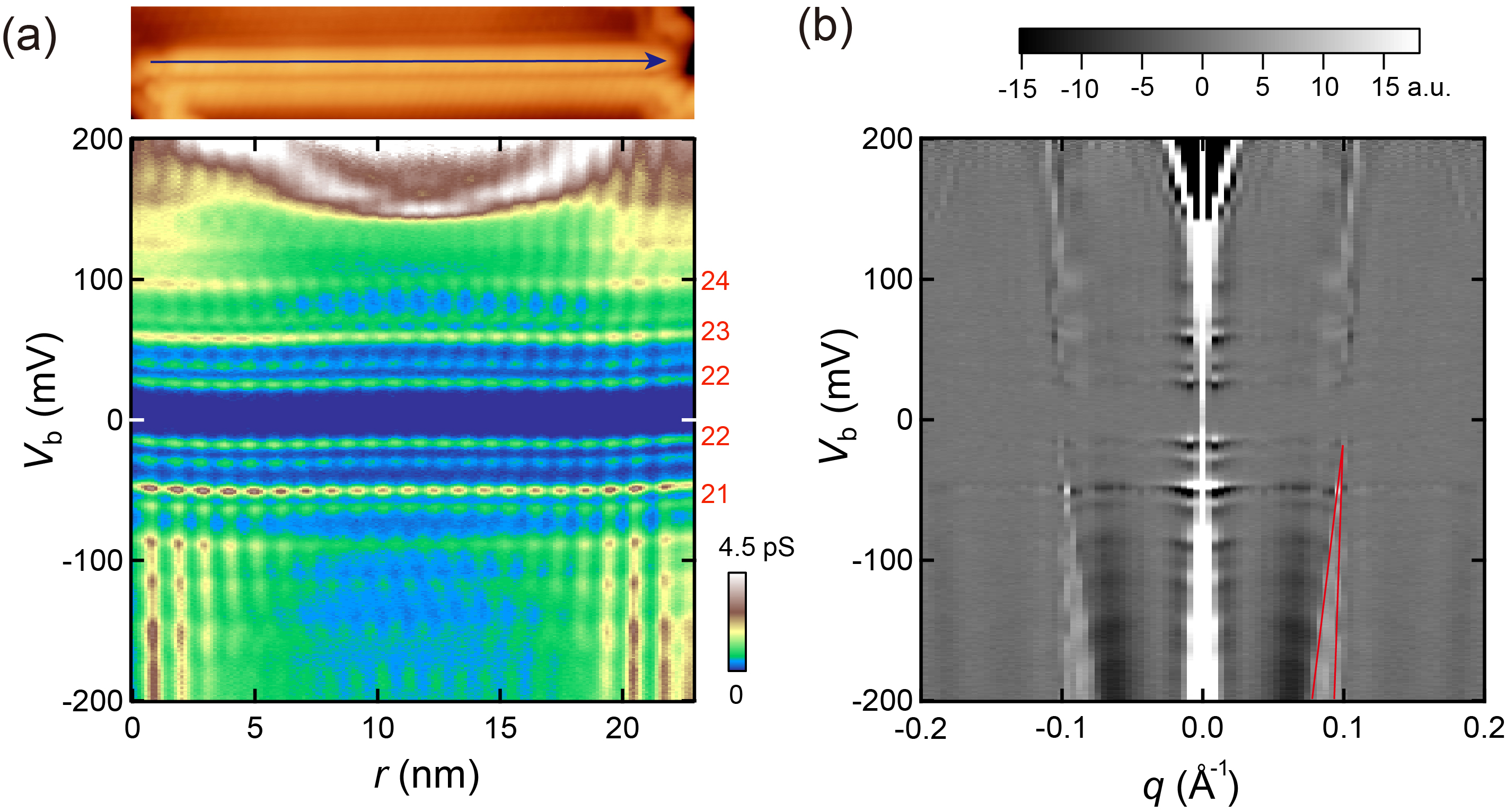


Figure S12. Node number labeling for discrete levels of MTB and spin-charge separation. (a) 2D conductance plot (bottom) [*V*_t_ = 200 mV, *I*_t_ = 100 pA, *V*_mod_ = 1.414 mV (rms)] of a type 2 MTB obtained along a black line shown in its STM image (top) (*V*_t_ = 500 mV, *I*_t_ =10 pA). Node numbers of the discrete levels are indicated with red numbers. (b) Fast Fourier transformation of the conductance plot of (a). There appear two separated branches of energy dispersions (marked with two red lines) below approximately 40 mV, which is a signature of spin-charge separation from TLL behavior.

**Supplementary References**

S1. Barja, S. *et al.* Charge density wave order in 1D mirror twin boundaries of single-layer MoSe_2_. *Nat. Phys.* **12**, 751-756 (2016).

S2. Hong, J. *et al*. Inversion domain boundary induced stacking and band structure diversity in Bilayer MoSe_2_. *Nano.Lett.***17** (11), 6653-6660 (2017).
